# Study of Candelilla Wax Concentrations on the Physical Properties of Edible Nanocoatings as a Function of Support Polysaccharides

**DOI:** 10.3390/polym15051209

**Published:** 2023-02-27

**Authors:** Claudia I. García-Betanzos, Humberto Hernández-Sánchez, Sergio A. Ojeda-Piedra, Araceli Ulloa-Saavedra, David Quintanar-Guerrero, María L. Zambrano-Zaragoza

**Affiliations:** 1 Laboratorio de Procesos de Transformación y Tecnologías Emergentes de Alimentos, Facultad de Estudios Superiores Cuautitlán, Universidad Nacional Autónoma de México, Cuautitlán Izcalli CP 54714, Mexico; 2 Laboratorio de Biotecnología de Alimentos, Escuela Nacional de Ciencias Biológicas, Instituto Politécnico Nacional, Ciudad de México CP 07738, Mexico; 3 Laboratorio de Investigación y Posgrado en Tecnologías Farmacéuticas, Facultad de Estudios Superiores Cuautitlán, Universidad Nacional Autónoma de México, Cuautitlán Izcalli CP 54745, Mexico

**Keywords:** nanocomposite films, xanthan gum, carboxymethyl cellulose, tensile properties, barrier properties, solid lipid nanoparticles

## Abstract

Solid lipid nanoparticles (SLN) based on candelilla wax were prepared using the hot homogenization technique. The resulting suspension had monomodal behavior with a particle size of 809–885 nm; polydispersity index < 0.31, and zeta potential of −3.5 mV 5 weeks after monitoring. The films were prepared with SLN concentrations of 20 and 60 g/L, each with a plasticizer concentration of 10 and 30 g/L; the polysaccharide stabilizers used were either xanthan gum (XG) or carboxymethyl cellulose (CMC) at 3 g/L. The effects of temperature, film composition, and relative humidity on the microstructural, thermal, mechanical, and optical properties, as well as the water vapor barrier, were evaluated. Higher amounts of SLN and plasticizer gave the films greater strength and flexibility due to the influence of temperature and relative humidity. The water vapor permeability (WVP) was lower when 60 g/L of SLN was added to the films. The arrangement of the SLN in the polymeric networks showed changes in the distribution as a function of the concentrations of the SLN and plasticizer. The total color difference (ΔE) was greater when the content of the SLN was increased, with values of 3.34–7.93. Thermal analysis showed an increase in the melting temperature when a higher SLN content was used, whereas a higher plasticizer content reduced it. Edible films with the most appropriate physical properties for the packaging, shelf-life extension, and improved quality conservation of fresh foods were those made with 20 g/L of SLN, 30 g/L of glycerol, and 3 g/L of XG.

## 1. Introduction

Edible films are thin layers of semi-solid materials that are assembled to improve the properties of fresh foods. They are generally recognized as safe (GRAS) [1]. The materials utilized to produce these films include polymers such as proteins and polysaccharides, whose function is to approach the barrier to O_2_ and CO_2_. However, they are poor barriers to water vapor. The lipids incorporated into these films form a hydrophobic barrier that reduces water vapor permeability, protects fruits and other foods from damage due to friction, and gives these edible coatings greater flexibility and cohesion [2]. The main function of these films when applied to fresh products is to delay maturation and the eventual senescence that occurs after harvesting. This is achieved by regulating the gas exchange, water vapor, moisture, and light, as well as preventing the volatilization of flavor compounds [3,4].

One of the polysaccharides most often used in the manufacturing of film-forming dispersions and thus edible coatings is XG, which is produced from the fermentation process of *Xanthomonas campestris*. XG is GRAS according to the FDA. Because it remains stable under changes in temperature and pH, it aids in obtaining emulsions that can reduce surface tension and ease the integration or addition of bioactive compounds as a controlled release system for edible coatings [5].

Recently, the demand for improving the functionality of edible films to meet consumer requirements has led to the development and application of novel materials that, due to their functional properties, extend food preservation for longer periods. This has given rise to the elaboration of nanocomposite films, which consist of a natural polymer matrix with added organic/inorganic fillers that have at least one magnitude on the nanometric scale [6]. Nanotechnology is commonly used to improve the properties of edible coatings by generating renewed materials that present enhanced mechanical, thermal, and water vapor barrier properties [7]. In this regard, García-Betanzos et al. [8] demonstrated that incorporating SLN made with Candeuba^®^ wax (mixture of *Euphorbia Cerifera* and *Copernicia Cerifera*) improved the mechanical and gas barrier properties of films when added to an XG matrix. In the same way, an increase in the shelf life was observed when guava fruit was coated with these systems [9]. The incorporation of SLN and protein into the mechanical properties of edible coatings has been studied. The observations showed that adding SLN decreased the WVT [10], whereas adding Tween 20 as an emulsifier agent in the elaboration of nanoparticles resulted in a reduction in tensile strength. Edible coatings prepared with lipids only do not have good mechanical properties and are weaker, but adding a lipid to films made with polysaccharides improves the water vapor barrier and increases the film’s strength, adhesion, and elastic properties [11].

SLN are aqueous colloidal dispersions, ranging in size from 40 to 1000 nm [12], which consist of lipids that are solid at room temperature. Hot homogenization is the most commonly used technique for producing SLN since this process does not require solvents other than water and is based on the formation of a nanoemulsion at high temperatures. Solid particles are expected to be formed by the cooling of the sample to room temperature or below. The surface area behavior of SLN is different from that of particles with a micrometric size, showing higher diffusion rates and better transport properties due to its low viscosity [13]. As mentioned above, SLN have been shown to enhance the properties of edible films and coatings. For this reason, this study was designed to analyze the impact of using candelilla wax as the lipid phase in SLN, as well as the effects of various polysaccharide types and plasticizer concentrations on the aforementioned material properties.

Candelilla wax (*E. antisyphilitica* Zucc.) is an endemic species found in the semiarid regions of northern Mexico and Texas. It is composed of 49–50% *n*-alkanes with 29–33% carbons, 20–29% high-molecular-weight esters, 12–14% alcohols and sterols, and 7–9% free acids [14,15]. It is considered one of the most effective waxes for blocking moisture migration [16]. The addition of candelilla wax to edible coatings was reported in the conservation of blackberry fruits by employing guar gum. This coating helped to increase the fruit´s shelf life. Candelilla wax and Arabic gum nanocoatings have also been developed as a vehicle for phytomolecules for use with tarbush. Products containing this substance were found to increase the shelf life of Golden Delicious apples during 8 weeks of refrigeration and 4 weeks of commercial storage at the industrial level [16]. However, some reports suggest that coatings employing candelilla wax in the dispersal phase are breakable due to the high melting point of this material (68.5–72.5 °C) while its mechanical and thermal properties and moisture barrier have not yet been studied in combination with polysaccharides such as XG and CMC.

The main aim of this work was to study a nanocomposite edible film by adding candelilla wax SLN to an XG and CMC continuous matrix in order to analyze its impact on the mechanical, optical, thermal, and barrier properties of films. Candelilla wax nanoparticles have never been used to strengthen edible films. The literature suggests that reducing particle size may have significant effects on the brittleness, color, and water vapor permeability due to the larger contact surface and hydrophobic nature of this material.

## 2. Materials and Methods

### 2.1. Materials

PVA-205 polyvinyl alcohol (m: 4.6–5.4 mPa·s) was used as the stabilizer (Sigma-Aldrich^®^, St. Louis, MO, USA). Xanthan gum derived from *Xanthomonas campestris* (Mw ≈ 2 × 10^−6^ g/mol and η_int_ = 7627 mL/g) and sodium carboxymethyl cellulose Cekol^®^ 30,000 (Mw: 240.20 g/mol) were used to form the film (CP Kelco, Mexico City, Mexico). Glycerol (98%) was used as the plasticizer agent (Droguería Cosmopolita, Mexico City, Mexico), whereas Candelilla REAL^®^ wax (melting point: 68–72.5 °C) purchased from Multiceras^®^ S.A. de C.V. (Monterrey, Mexico) was used as the solid lipid. Distilled water was acquired from Milli-Q equipment (Millipore^®^ Corporation, Bedford, MA, USA). All other reagents were analytical grade.

### 2.2. Solid Lipid Nanoparticle Preparation

The SLN were prepared using both a lipid and an aqueous phase. The first phase consisted of melting 100 g/L of candelilla wax at 90 °C while a PVA-205 solution (50 g/L) was heated up to the same temperature. Both phases were homogenized using the high-shear stirring technique (Ultra-Turrax^®^ T5; KikalborTechnik, Germany, with an S25N-25G, IKA^®^ disperser element) during 3 to 5 min cycles at 2094.4 s^−1^. The solidification of the SLN occurred during the cooling of the suspension at 25 °C [16,17].

### 2.3. Dynamic Light Scattering (DLS)

A Z-sizer 4 (Zetasizer Nano Series, Malvern Ltd., Enigma Business Park, Grovewood Road, Malvern, UK) was employed to determine the particle size (PS), polydispersion index (PDI), and zeta potential (ζ). The evaluation was carried out for pure candelilla wax SLN (100 g/L) for 5 weeks. The PS and PDI were measured using a laser light-scattering technique at a 90° fixed angle and a temperature of 25 °C. The zeta potential was evaluated by electrophoretic movement with a Z-sizer 4 at 90 ° (Zetasizer Nano Series). The dilutions were performed with Milli-Q^®^ water and the results obtained were normalized via polystyrene standard dispersion (ζ = −55 mV). All measurements were taken in triplicate at room temperature.

### 2.4. Film-Forming Dispersions

The edible film-forming dispersions were prepared by dispersing 3 g/L of each polysaccharide in distilled water using a stirrer (Eurostar Power Control Visc, IKA^®^ Werke, Staufen, Germany) at room temperature for 10 min and a speed of 104.72 s^−1^. Subsequently, two concentrations of SLN (20 and 60 g/L) were added to study their impact on the mechanical, optical, and barrier properties, as well as the thermal behavior and film microstructure. Two concentrations of plasticizer (10 or 30 g/L) were added to the film dispersions.

### 2.5. Film Formation

Once the dispersions were obtained, 22.5 mL of each was cast in a Teflon plate (192.5 cm^2^) with circular geometry. The dispersions were stored for 24 h in an acrylic chamber at a temperature of 35 °C and 60% of RH to allow them to dry. The thickness was evaluated with a digital micrometer (Mitutoyo Model 293-348, Kanagawa, Japan) with an error of 0.001 mm. To calculate the thickness, 9 measurements taken at different positions of the film were used. All films were manufactured following the same method to ensure the variations were a function of the storage conditions and formulations only. The formulations tested are shown in Table 1.

### 2.6. Water Vapor Permeability (WVP)

The WVP was evaluated at 10, 25, and 35 ± 2 °C using the ASTM E96/E96M-05 method with minor variations. Briefly, 3 g of silica gel was added to glass vials to ensure 0% of RH within each container. Subsequently, each vial was covered with films of a 1.5 cm diameter. The samples were conditioned in acrylic chambers using saturated salt solutions (NaBr–60% RH; NaCl–70% RH; and KCl–85% RH). The weight variations of the vials were documented over a period of 205 h. Each determination was evaluated in triplicate. The film previously determined thickness was utilized for all WVP estimations.

### 2.7. Mechanical Properties

A Brookfield texture analyzer (TA-CT3, Brookfield Engineering Laboratories, Inc., Middleboro, MA, USA) was utilized to evaluate the mechanical properties of the films using the ASTM D882-02 method. Before performing the tests, the films were cut into rectangular shapes (10 mm wide × 40 mm long) and conditioned at 60, 70, and 85% of RH and 10, 25, or 35 °C for 48 h. After this time, the mechanical properties were evaluated using the TA-DGF accessory (Dual-Grip Fixture for tensile testing of thin films or integrity of seals for packaging). The crosshead speed was set at 50 mm/min. The tensile strength (TS), elongation at breaking (E), and Young’s modulus (YM) were obtained. All assays were performed in triplicate at room temperature.

### 2.8. Whiteness Index of Films

The optical properties of the films were determined with an Agrocolor^®^ colorimeter (Apollinaire Ltd., Agrotechnology, Serqueux, France), and the R (red), B (blue), and G (green) parameters were obtained, which were then converted to L*, a*, and b* coordinates (L*: lightness; a*: red-green; b*: yellow-blue). The films’ color determinations were contrasted against a blank and both sides of the film were evaluated in duplicate to calculate the total color difference (Δ*E*). Determining the whiteness index (*WI*) did not require any background and was calculated as follows:

(1) $$∆E=\sqrt{{∆L}^{*2}+{∆a}^{*2}+{∆b}^{*2}}$$

(2) $$WI=100-\sqrt{\left(100-L^{*2}\right)+\left(a^{*2}\right)+(b^{*2})}$$

where ΔL*, Δa*, and Δb* represent the variations in the color parameters for the SLN–polysaccharide films vs. the white tile (*L* = 97.75, *a* = −0.49, *b* = 1.96) [9,18,19].

### 2.9. Differential Scanning Calorimetry (DSC)

The thermal properties of the bulk components, pure SLN, and non-conditioned edible films were evaluated using a Diamond Differential Scanning Calorimeter (Perkin Elmer Instruments, Waltham, MA, USA). For this technique, 10 mg of each specimen was precisely weighed in a 40 μL aluminum pan and then hermetically sealed. The heating tests were staged from −20 to 150 °C (10 °C/min). An empty aluminum pan was utilized as a blank and each thermograph was baseline-corrected.

### 2.10. Scanning Electron Microscopy (SEM)

Morphological analyses of the prepared films with different concentrations of SLN, glycerol, and polysaccharides were conducted using a high-resolution, cold-field scanning electron microscope (SEM) (Hitachi, SU-8230, Tokyo, Japan) with a BSE + BSE (U) detector, an acceleration voltage of 2.5 kV, and an average deceleration mode of 15 kV. The emission current was 5 Å with a working distance of 3.7 mm.

### 2.11. Statistical Analysis

An analysis of variance (ANOVA) was carried out to evaluate the effect of the formulation, temperature, and RH. All statistical differences between batches were analyzed with a Tukey test. The responses evaluated were the film thickness, tensile strength (TS), elongation at breaking (E), Young’s modulus (YM), total color difference (ΔE), and whiteness index (WI). Minitab^®^ (Minitab^®^ Statistical Software 17 Inc., Centre, PA, USA) software was used to carry out the analysis of variance.

## 3. Results and Discussion

### 3.1. Characterization of the SLN

Table 2 shows the results of the initial SLN suspension (100 g/L) from which dilutions were prepared to produce the edible films. The PS ranged from 809 to 885 nm and showed a slight increase with respect to the storage time. However, the ANOVA did not show any statistical differences (*p* > 0.05). The candelilla wax SLN were larger than others previously obtained by our group. This increase in size can be attributed to the composition of the candelilla wax, which, unlike carnauba wax, for example, has a high hydrocarbon content (50% vs. 2–3% of carnauba wax [14]). A study by García-Betanzos et al. [8] reported a smaller particle size (240–261 nm) after analyzing a similar SLN suspension (100 g/L) prepared with Candeuba^®^ wax (mixture of candelilla and carnauba waxes) and Pluronic F-127 as a surfactant. According to their results, the candelilla wax presented a larger chain (50–57% weight) than the Candeuba^®^ wax (30–34% weight). Another study using Beeswax SLN reported a particle size of 214–227 nm when polyvinyl alcohol was used as a stabilizer. This was considerably smaller than the size of the candelilla wax SLN. Gonçalves et al. 2022 [20] observed that using 3% of beeswax to produce SLN contributed to the reduction in the particle size. Mäder and Mehnert [21] confirmed the influence of the solid lipid used on the particle size by highlighting that the use of shorter fatty acid-chain lipids reduced this parameter. To reduce the particle size, nanostructured lipid carriers made of candelilla wax with jojoba oil were prepared. Although the reported particle size was ~100 nm, the carriers did not exhibit Gaussian behavior and the percentage of candelilla wax was not reported [16]. The results showed that the particle size was affected by the applied shearing force, wax concentration, and presence of other stabilizing components or integrated oils in the mixtures.

In our study, the PDI was <0.31 throughout the storage time, suggesting that the particle size distribution in the system was narrow. A PDI value between 0.1–0.4 indicated that the system had a moderate particle size distribution [22]. The ANOVA indicated no statistically significant differences (*p* > 0.05) among the PDI values obtained during the 5 weeks, although the ζ increased significantly (*p* ≤ 0.05) during the storage, with a value of −3.5 mV at week 5. This indicates that the polyvinyl alcohol used as the surfactant was unable to provide a “strong” electric field around the SLN. However, the fact that upon associating the ζ with the PS, no significant changes were observed, leading to the conclusion that the system stability was due to the steric effect provided by the polyvinyl acetate functional groups, which can prevent particle agglomeration. Steric repulsive forces between electrons resulted in structured groups of molecules stabilized in the way that opposites attract and charges repel. According to Gordillo-Galeano and Mora Huertas [23], the ζ is influenced by the particles’ surface properties and depends on the environment in which they are dispersed, as well as the concentration of the sample used for the evaluation. The ionic strength, pH, nature of the solvent, and content of the electrolytes in the solution impact the ζ values and, therefore, the sign of the charge shown by the nanoparticles.

### 3.2. Thickness and Water Vapor Permeability (WVP)

Owing to the hydrophilic nature of polysaccharides and glycerol, we expected to find changes in the water adsorption of the films due to the composition, temperature, and relative humidity. The results for the thickness and WVP are shown in Table 3. In all cases, adding 60 g/L of SLN yielded films that were 65% thicker (*p* ≤ 0.05) than those to which only 20 g/L were added, although an identical volume of film-forming dispersion was cast. When zein protein films were reinforced with TiO_2_-SiO_2_ core-shell nanoparticles, thicker films were obtained. These changes can be attributed to the fact that the film-forming dispersions contained more dry matter at higher SLN concentrations [24]. A study in which specific concentrations (0–60%) of palm wax were added to fish gelatin films reported an increase in film thickness as a consequence of a higher wax concentration and cited the consideration that a greater interaction among the components occurs [25]. In our study, the use of 30 g/L of glycerol affected (*p* ≤ 0.05) the thickness because the films formulated with this amount of plasticizer were 15.6% (60 g/L SLN) and 5% (20 g/L) thicker, respectively, than when only 10 g/L was added. The use of the plasticizer showed that polysaccharide films become more hydrophilic with increases in the plasticizer concentration due to the high hygroscopicity of the reagent [26]. This effect was also detected in our work. In addition, we found that regardless of the moisture content, the increase in temperature caused a decrease in the aw of the films due to the increase in unbounded water molecules from the film structure, thereby decreasing the free energy of the system. However, this modification of the hydrophilic bonds allows the film to adsorb higher moisture from the environment, thereby increasing its thickness [27,28] (see Table 3). A higher RH increase caused a higher vapor pressure, facilitating the adsorption of the water molecules and resulting in a large amount of moisture observed in the films.

Table 3 shows the results for the WVP. The films with 60 g/L of SLN but different glycerol concentrations added showed the lowest (10 g/L) and highest (30 g/L) WVP (10 °C: all RH; 25 °C: RH of 60 and 70%; 35 °C: RH of 60%). These findings suggest that a high concentration of submicron-sized particles effectively reduced the water vapor barrier in the films. The plasticizer is the ingredient in edible films that has the greatest impact on the permeability since it breaks up intermolecular bonding, facilitating the diffusion of water, and increases moisture transfer through the hydrophilic portion of the films [11,29]. In addition, it has been reported that crystalline particles (as in the case of SLN) decrease the hygroscopicity in polysaccharide films because moisture transfer occurs preferentially through the non-crystalline areas. However, for films with 30 g/L of glycerol added, the higher affinity of the plasticizer to the polysaccharide compared to the SLN produced nanoparticle agglomeration that generated continuous matrix spaces that lacked any hydrophobic effect and showed greater WVP [10]. Films with 20 g/L of SLN added had WVP values that were low but statistically significantly different (*p* ≤ 0.05) from those of the films with the best barrier properties. We also observed that high glycerol concentrations negatively affected the WVP (10 °C: RH of 60 and 70%; 25 °C: RH of 70%; and 35 °C: RH of 60 and 70%).

The conditioning of the films at different temperatures also had an impact (*p* ≤ 0.05) on WVP values. Counter to our expectations, in most cases, an increase in temperature reduced the WVP. This behavior can be attributed to the possible water saturation of the film structure caused by its exposure to conditions that highly promoted water diffusion (temperatures ≥ 25 °C). In this case, it appears that water saturation reduced the interstitial spaces in the film, thus decreasing the diffusion rate of water molecules [30]. Regarding the RH, an 85% increase generated higher WVP values, although this behavior was only observed at temperatures above 25 °C. The mobility of water molecules decreased at low temperatures, causing the non-uniform trend of the WVP observed at 10 °C. The nanometric scale of the SLN improved the WVP and reduced the thickness compared to the candelilla wax–mesquite gum films, which had reported thicknesses of 0.199–0.420 mm and WVP values of 3.93–9.48 g mm/h m^2^. These figures were significantly higher than those reported in Table 3 [31], suggesting a better interaction in the network formed with the studied polysaccharides when the wax was incorporated in nanometric sizes.

### 3.3. Mechanical Properties

The most representative mechanical properties of edible films are the TS and E. The TS is the ability of a film to prevent rupture when it is subjected to tensile stress and is expressed in MPa. The elongation at breaking (E) is the length to which a material can be stretched before it breaks and is expressed as a percentage. These mechanical properties are important because they give us an idea of the behavior that films and coatings will exhibit when they are applied to food, as well as the changes associated with external factors such as temperature, RH, and film composition [32]. Table 4 shows the influence of the temperature, RH, and film composition on these mechanical properties. The TS of the films ranged from 0.325–20 MPa. The formulations with 60 g/L of SLN, 10 g/L glycerol, and 3 g/L XG showed the greatest (*p* ≤ 0.05) resistance to breakup, suggesting that the addition of high amounts of SLN can produce stronger films. This finding can be attributed not only to the geometry and rigidity of the particles but also to the development of a stiff continuous network of SLN throughout the polysaccharide matrix. Studies with palm wax- and gelatin-based films have reported that an increase in the wax content of up to 15% increases the TS from 39 to 62% [25]. In our study, the most significant increase in the TS was found to be 85% for films prepared with 60 g/L of SLN-XG and 10 g/L of glycerol, whereas the lowest increase was found for SLN with the addition of 60 g/L CMC and 30 g/L of glycerol. No statistically significant differences (*p* > 0.05) were observed for the plasticizer content.

The high YM and low E observed in the formulations of these films suggest a lack of flexibility. A study of starch-based films found that the Young’s modulus and TS at the breaking point were greater when the plasticizer content was low and that the elongation decreased [18]. The films made in this study precisely mimicked this behavior since those with the addition of 60 g/L of SLN, 30 g/L of plasticizer, and 3 g/L of CMC showed a low TS and YM and a high E. The results shown in Table 4 suggest that the SLN reinforced the edible films without significantly affecting the elasticity. According to the WVP results, this formulation showed the lowest barrier properties, which suggests that the water vapor absorbed during conditioning functioned as a plasticizer. The volume of water absorbed across the edible films enhanced the polyol content [31] and since water is a low-molecular-weight component, it increased the free volume, allowing greater backbone chain segmental mobility [31].

Reducing the SLN concentration (20 g/L) improved the TS by 89% in the films with the addition of 30 g/L of glycerol and 3 g/L XG and by 67% in the films with the addition of 10 g/L of glycerol and 3 g/L CMC. This confirms that the SLN concentration is the main factor that affects the films’ strength, followed by the plasticizer. The YM obtained in the first case was in the interval of 0.010–0.054 MPa, whereas in the second case, it was 0.118–0.534 MPa. The E values ranged from 39 to 93.3% and 22 to 34.54%, respectively. The increase in the flexibility of the films according to the plasticizer content may be associated with the modifications to the structure of the polymeric matrix, which became less dense, such that applying tension facilitated the movements of the polymer chains [9]. In most cases, increases in temperature (10–25 °C) and RH (60–70%) during conditioning decreased (*p* ≤ 0.05) the rigidity but improved the elasticity of the films. This effect, however, was not observed when the temperature was raised to 35 °C with the RH at 35%. The addition of 20 g/L of SLN, 10 g/L of glycerol, and 3 g/L of CMC did not generate any statistically significant differences with respect to temperature (*p* > 0.05).

### 3.4. Color

Table 5 shows the color properties of the films as a function of the temperature, RH, and film composition, which are expressed as ΔE and WI. In agreement with the results of the ANOVA, no statistically significant differences in the ΔE were found with regard to the SLN content when the films were stored at 10 °C, but a statistically significant difference (*p* ≤ 0.05) between the batches was found as the temperature increased, suggesting that temperatures above 25 °C modify the visual appearance of the films (*p* ≤ 0.05). The composition and characteristics of the films with respect to the ΔE can be attributed to the nature of the waxes, which can change from a yellowish color to white, as well as the stabilizers used [25]. Despite this, the formulations that showed the highest ΔE values at all temperatures were those containing the maximum concentration of SLN (60 g/L). This behavior can be explained by the greater amount of lipid nanoparticles dispersed in the polymeric network since the SLN were made of candelilla wax, which tends to transfer its yellowish coloration to the films.

When only 20 g/L of SLN was added, the ΔE values were 23 and 24% lower for the films with 10 g/L and 30 g/L of plasticizer added, respectively. The color of the films can also be affected by the thickness. For example, the thicker films prepared in this study (101–252 µm), which corresponded to the incorporation of 60 g/L of SLN, had a more yellowish appearance (higher positive b values). The ΔE values provided us with a clearer analysis of the films’ color characteristics since they involved the three color parameters: lightness (L), red-green hue (a), and yellow-blue shade (b) [8], and reflected the difference between the two colors. A study of edible films prepared with whey protein and candelilla wax reported yellowing as the ΔE reported was higher than the value obtained in our study. The optimal conditions for the edible films in terms of color in that study were 0.16% candelilla wax, 0.54% glycerol, and 1% whey protein [33].

Increasing the proportion of submicron-sized candelilla wax markedly reduced the color difference. All films stored at 10 °C showed an appreciable color difference (3–6), but increasing the temperature increased this difference (6–12) in the films with 60 g/L of SLN added. The RH did not show any statistically significant differences (*p* > 0.05) in the total color analysis. No statistically significant differences in the WI were found according to the SLN content when the films were stored at temperatures ≤ 25 °C, but a temperature increase modified this behavior so we were able to establish that conditioning the films under mild heat (35 °C) impacted (*p* ≤ 0.05) their physical appearance and would, therefore, have an effect on consumer acceptance. Contrary to the ΔE results discussed previously, the films with a higher WI contained a concentration of 20 g/L of SLN, suggesting that the samples with the smaller number of submicron-sized particles maintained transparency better than the control films made only with polysaccharides (data shown in Section 2.8) due to the absence of particles with a yellowish coloration that had slight selective absorption. Adding 60 g/L of SLN decreased the WI (although the difference was not statistically significant at temperatures below 25 °C (*p* > 0.05)). This behavior is undesirable in edible films because when applied, it alters the product’s appearance.

### 3.5. Differential Scanning Calorimetry (DSC)

**Figure 1:**
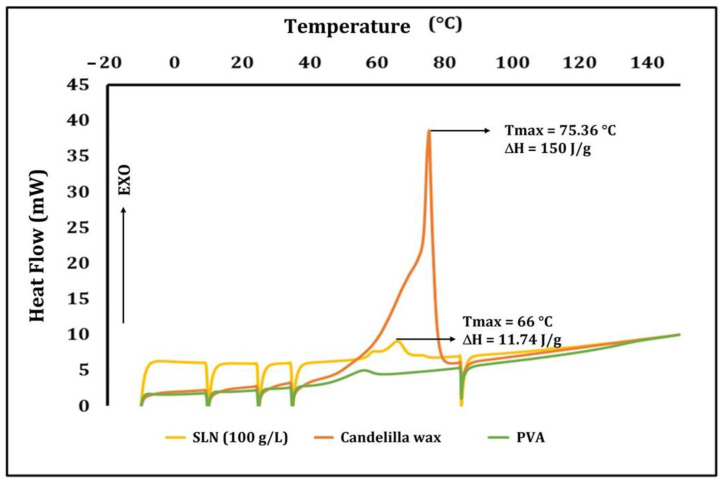
Thermal behavior of raw materials used to form SLN dispersions (100 g/L).

Figure 1 illustrates the thermal behavior of the candelilla wax and PVA used to prepare the SLN and the SLN suspension (100 g/L) from which the dilutions were made to reach the concentrations added to the films. The surfactant showed an endothermic peak (Tmax) at 55.76 °C, a thermal transition that was not characteristic of the compound and may represent the fusion of some impurities or crystals not usually found in this material. It has been reported that the melting temperature of PVA is 237 °C, but because our tests were performed at a temperature below this, it was impossible to observe the transition. The lipid exhibited a Tmax of 75.36 °C, which is characteristic of candelilla wax fusion, mainly of the *n*-alkanes that comprise the major component of this compound [34]. Recent characterizations of different sources of candelilla wax have reported a melting point of 66 °C with an ΔH of 111.85–141.52 J/g [15]. The heat required (ΔH) to achieve the phase transition was 150 J/g. This parameter was a function of the highly crystalline nature of the wax. However, the variations in the melting point and ΔH can be attributed to the origin and purity of each ingredient. Previously, García-Betanzos et al. [8] reported the melting point of hydrated xanthan gum and CMC polysaccharides as 130.4 °C and 227 °C, respectively, whereas glycerol has been shown to degrade at 178 °C.

Figure 1 also shows the calorimetric assay of the SLN suspension (100 g/L), which showed a Tmax of 66 °C. This reduction in the melting temperature compared to the pure wax is due to the interaction of the lipid with the other ingredients that form the suspension, mainly the surfactant. Similarly, the reduction in the crystallinity due to these interactions lowered the ΔH required to carry out the transition (11.74 J/g). The reduction in the Tmax and ΔH was produced by the decrease in the chemical potential caused by adding the surfactant, which interacted with water to allow a plasticizer effect that reduced the heat required to modify the system phase. The reduction in the particle size (nm) also contributed to this behavior. García-Betanzos et al. (2016) [8] reported a Tmax of 75.3–107 °C for SLN (100 g/L) made of Candeuba^®^ wax; however, their particles had a size of ~227 nm, whereas the average particle size reported in our study was 850 nm.

**Figure 2:**
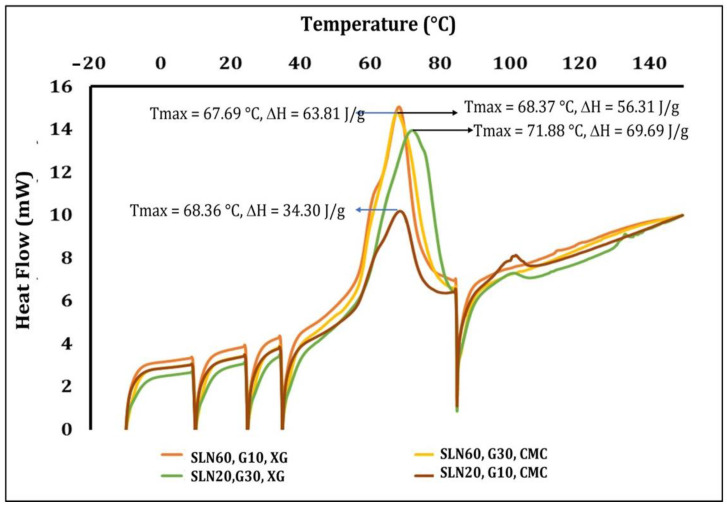
Thermal behavior of films with SLN added at different concentrations.

Figure 2 shows the thermograms of the films with SLN added at different concentrations. The formulation with the highest Tmax contained 60 g/L of SLN, 10 g/L of glycerol, and 3 g/L of XG (71.88 °C). The other formulations showed a Tmax of 67.69–68.37 °C. The ΔH was higher for both films with the maximum concentration of SLN added, but adding 30 g/L of plasticizer caused a greater decrease in this parameter (63.81 J/g) compared to adding only 10 g/L (69.69 J/g). The samples with 20 g/L of SLN added presented a ΔH of 34.30 J/g (30 g/L of glycerol) and 56.51 J/g (10 g/L). It has been suggested that increasing the glycerol content reduces the Tmax and ΔH in films made of fish gelatin starch, suggesting that the plasticizer decreases the intermolecular forces and improves polymeric-chain flexibility [35]. The behavior observed with the change in the ΔH was in contrast with the mechanical properties of films observed previously, where low ΔH values were associated with more flexible and elastic films.

### 3.6. Scanning Electron Microscopy (SEM)

**Figure 3:**
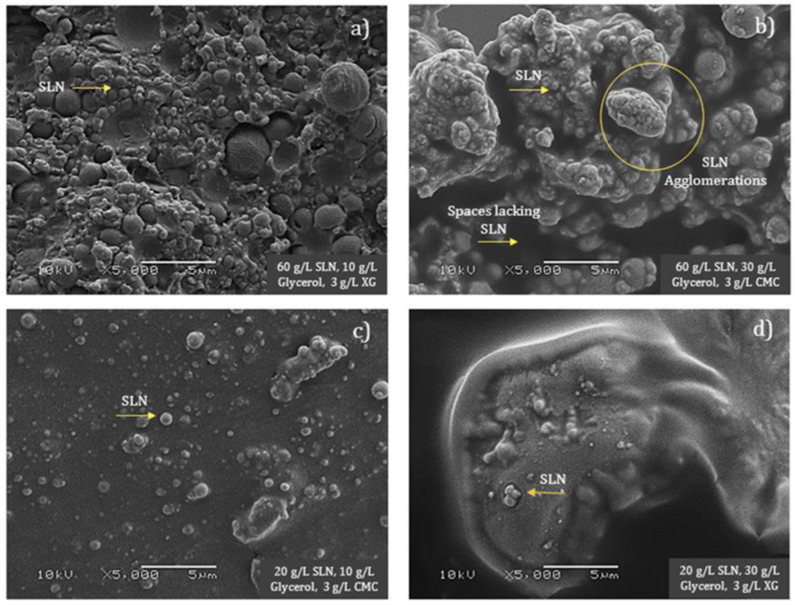
Microscopic surfaces of edible films prepared with 60 g/L of SLN, 10 g/L of glycerol, XG (**a**); 60 g/L of SLN, 30 g/L of glycerol, CMC (**b**); 20 g/L of SLN, 10 g/L of glycerol, CMC (**c**); and 20 g/L of SLN, 30 g/L of glycerol, XG (**d**).

Figure 3 illustrates the microscopic surfaces of the edible films. Figure 3a,b depict the films with the maximum concentration of SLN added. As mentioned above, in both cases, the observations showed a large number of submicronic particles distributed along the continuous matrix, although in the films with the addition of 30 g/L of glycerol and 3 g/L of CMC (b), the presence of the SLN agglomerates was clear. This phenomenon may be due to the physical interaction between the plasticizer and polysaccharide due to the affinity of these materials (hydrophilic nature). This may trigger lipid-to-lipid interactions (clusters) in some areas of the surface and the formation of empty spaces without hydrophobic effects in others through which the water of the conditioning atmosphere diffused to produce films with a poor water vapor barrier. This effect was not seen when only 10 g/L of glycerol was added (a), where a homogeneous distribution of SLN in the polymeric matrix was found that supported the WVP and the results found for the mechanical properties. In contrast, Figure 3c,d show the microstructure of the edible films with 20 g/L of submicron particles added, where a lower concentration of particles homogeneously distributed in the polymeric matrix was observed. Comparing these samples with those containing the maximum concentration of SLN revealed structural modifications in the polymeric matrix as the network reduced its density. This effect impacted the mechanical, optical, and barrier properties by providing good elasticity (low YM, high E), reducing the tensile strength of the films, and enhancing the hydrophobic influence of the submicron particles, all of which contribute to low WVP values. Finally, the color of these films was less affected so they maintained greater transparency and had less effect on the physical appearance of the product when applied.

## 4. Conclusions

The SLN formulated in this work remained sterically stable for 5 weeks due to the use of PVA as a surfactant compound. The incorporation of candelilla wax SLN into the XG and CMC networks improved the mechanical properties of the films since the geometry and rigidity of the particles caused changes in stiffness, elasticity, and flexibility. Likewise, the natural crystallinity of the SLN modified the hygroscopicity of the films and the structural arrangement of the continuous matrices, thus enhancing the water vapor barrier properties. However, the addition of submicron-sized candelilla wax particles resulted in a yellowish coloration of the films, which was more pronounced when 60 g/L of SLN was added. This directly impacted the ΔE and WI parameters. The best results were observed in the formulations with 20 g/L of SLN added, especially those containing 30 g/L of glycerol and 3 g/L of XG, as the microstructural analysis showed a better distribution of nanoparticles along the matrix that resulted in a low WVP. Adding 30 g/L of plasticizer decreased the intermolecular forces and improved the mobility of the polymeric chain, resulting in low ΔH values and, therefore, films that were more flexible and elastic that can maintain their physical structure by avoiding deformations and ruptures when applied to fresh foods. In conclusion, candelilla wax is a good option for preparing nanocomposite films since its interaction with the polysaccharide used as the continuous matrix improved the properties of the films. Most nanocomposites are eco-friendly, novel materials, as is the case with the nanocoating developed and studied by our group. These characteristics, together with the improvements in the mechanical, water vapor barrier, and structural properties, allow nanocoatings based on SLN to preserve the shelf life of whole fruits. Climacteric fruits, which are characterized by a high respiration rate, can be a good option to experiment “*in vivo*” with the conservative effect of these nanocoatings since their excellent barrier properties can have a sluggish effect on the respiration rate, thus delaying ripening and senescence. We suggest the application of the developed nanocoatings to fruits such as pears, apples, guavas, tomatoes, plums, and figs.

## Figures and Tables

**Table 1:** Components used in the formation of the films.

Nomenclature	Component			
	SLN (g/L)	Glycerol (g/L)	XG (g/L)	CMC (g/L)
SLN60, G10, XG	60	10	3	-
SLN20, G30, XG	20	30	3	-
SLN60, G30, CMC	60	30	-	3
SLN20, G10, CMC	20	10	-	3

**Table 2:** Particle size (PS), polydispersity index (PDI), and zeta potential (ζ) of the SLN.

Time (Week)	Particle Size (nm)	PDI	ζ (mV)
0	809.7 ± 53.7 ^a^	0.08 ± 0.04 ^a^	−18.8 ± 0.7 ^a^
1	829.6 ± 25.6 ^a^	0.10 ± 0.05 ^a^	−14.2 ± 0.5 ^b^
2	866.4 ± 39.7 ^a^	0.12 ± 0.07 ^a^	−8.7 ± 0.8 ^c^
3	879.6 ± 26.3 ^a^	0.17 ± 0.13 ^a,b^	−7.9 ± 0.2 ^c^
4	882.8 ± 25.3 ^a^	0.24 ± 0.07 ^a,b^	−5.7 ± 1.2 ^c,d^
5	885.9 ± 68.5 ^a^	0.31 ± 0.02 ^a,b^	−3.5 ± 2.4 ^c,d^

Each value represents the mean ± standard deviation of three replicates. Different letters indicate statistically significant differences (*p* ≤ 0.05) between weeks at 25 °C.

**Table 3:** Thicknesses and WVP of edible films under different storage conditions.

	Thickness (mm)	WVP (g × mm/m^2^ × h × kPa)	Thickness (mm)	WVP (g × mm/m^2^ × h × kPa)	Thickness (mm)	WVP (g × mm/m^2^ × h × kPa)
Formulation	60% RH		70% RH		85% RH	
	10 °C					
SLN60, G10, XG	0.119 ± 0.01 ^b,a^	0.483 ± 0.04 ^a,a^	0.101 ± 0.01 ^c,b^	0.418 ± 0.01 ^b,a^	0.097 ± 0.01 ^b,b^	0.166 ± 0.01 ^c,b^
SLN20, G30, XG	0.057 ± 0.01 ^b,b^	0.782 ± 0.01 ^a,b^	0.062 ± 0.01 ^b,a^	0.805 ± 0.01 ^a,a^	0.055 ± 0.01 ^c,b^	0.638 ± 0.01 ^a,c^
SLN60, G30, CMC	0.120 ± 0.01 ^b,b^	1.227 ± 0.11 ^a,b^	0.160 ± 0.01 ^a,a^	1.896 ± 0.01 ^a,a^	0.113 ± 0.01 ^c,b^	1.116 ± 0.01 ^a,b^
SLN20, G10, CMC	0.057 ± 0.00 ^b,b^	0.679 ± 0.02 ^a,c^	0.061 ± 0.01 ^a,a^	0.740 ± 0.01 ^a,b^	0.050 ± 0.01 ^b,c^	0.946 ± 0.03 ^a,a^
	25 °C					
SLN60, G10, XG	0.122 ± 0.01 ^b,b^	0.134 ± 0.01 ^b,c^	0.131 ± 0.01 ^b,ab^	0.233 ± 0.01 ^c,b^	0.146 ± 0.02 ^a,a^	0.554 ± 0.01 ^a,a^
SLN20, G30, XG	0.058 ± 0.01 ^b,c^	0.209 ± 0.01 ^b,b^	0.064 ± 0.01 ^b,b^	0.342 ± 0.01 ^b,a^	0.071 ± 0.01 ^b,a^	0.361 ± 0.01 ^b,a^
SLN60, G30, CMC	0.137 ± 0.01 ^a,b^	0.581 ± 0.02 ^b,c^	0.143 ± 0.02 ^b,ab^	0.788 ± 0.03 ^b,b^	0.160 ± 0.01 ^b,a^	0.883 ± 0.02 ^b,a^
SLN20, G10, CMC	0.066 ± 0.01 ^a,b^	0.263 ± 0.06 ^b,b^	0.064 ± 0.00 ^a,b^	0.315 ± 0.01 ^b,ab^	0.075 ± 0.01 ^a,a^	0.379 ± 0.01 ^b,a^
	35°C					
SLN60, G10, XG	0.137 ± 0.01 ^a,b^	0.096 ± 0.01 ^b,b^	0.150 ± 0.01 ^a,ab^	0.521 ± 0.02 ^a,a^	0.164 ± 0.02 ^a,a^	0.496 ± 0.01 ^b,a^
SLN20, G30, XG	0.063 ± 0.01 ^a,c^	0.208 ± 0.01 ^b,a^	0.068 ± 0.01 ^a,b^	0.212 ± 0.01 ^c,a^	0.081 ± 0.01 ^a,a^	0.203 ± 0.01 ^c,a^
SLN60, G30, CMC	0.142 ± 0.01 ^a,c^	0.443 ± 0.03 ^b,b^	0.151 ± 0.01 ^ab,b^	0.494 ± 0.01 ^c,b^	0.252 ± 0.01 ^a,a^	0.663 ± 0.03 ^c,a^
SLN20, G10, CMC	0.056 ± 0.01 ^b,c^	0.182 ± 0.01 ^b,b^	0.061 ± 0.01 ^a,b^	0.201 ± 0.01 ^c,ab^	0.077 ± 0.01 ^a,a^	0.211 ± 0.01 ^c,a^

Each value represents the mean ± standard deviation of three replicates. Different letters indicate statistically significant differences (*p* ≤ 0.05); the first letter corresponds to the analysis based on the temperature and the second to the RH of the storage.

**Table 4:** Mechanical properties of edible films under different storage conditions.

	TS (MPa)	YM (MPa)	E (%)	TS (MPa)	YM (MPa)	E (%)	TS (MPa)	YM (MPa)	E (%)
Formulation	60% RH			70% RH			85% RH		
	10 °C								
SLN60, G10, XG	8.01 ± 0.25 ^a,b^	5.72 ± 0.75 ^b,b^	4.03 ± 0.10 ^a,b^	7.58 ± 0.99 ^a,b^	6.73 ± 0.95 ^b,b^	3.71 ± 0.16 ^ab,b^	20.09 ± 1.49 ^a,a^	9.22 ± 0.27 ^a,a^	6.28 ± 0.31 ^a,a^
SLN20, G30, XG	1.17 ± 0.22 ^a,b^	0.02 ± 0.00 ^b,c^	59.12 ± 2.10 ^a,b^	0.75 ± 0.02 ^a,b^	0.03 ± 0.00 ^a,a^	82.07 ± 3.44 ^a,a^	1.80 ± 0.19 ^a,a^	0.03 ± 0.00 ^a,b^	53.21 ± 5.35 ^b,b^
SLN60, G30, CMC	2.28 ± 0.51 ^a,a^	1.13 ± 0.05 ^a,a^	17.00 ± 3.37 ^a,b^	2.04 ± 0.11 ^a,a^	1.12 ± 0.09 ^a,a^	24.39 ± 3.81 ^a,b^	2.47 ± 0.24 ^a,a^	0.60 ± 0.07 ^b,b^	49.05 ± 1.96 ^a,a^
SLN20, G10, CMC	2.11 ± 0.18 ^a,a^	0.30 ± 0.04 ^b,a^	24.49 ± 1.78 ^a,b^	1.64 ± 0.33 ^a,ab^	0.18 ± 0.00 ^ab,a^	24.64 ± 2.92 ^ab,b^	1.16 ± 0.12 ^b,b^	0.27 ± 0.06 ^b,a^	31.701 ± 1.57 ^a,a^
	25 °C								
SLN60, G10, XG	5.63 ± 0.72 ^b,a^	7.78 ± 0.92 ^b,a^	4.07 ± 0.37 ^a,ab^	5.07 ± 0.038 ^b,a^	6.57 ± 0.88 ^b,ab^	3.37 ± 0.63 ^b,b^	3.56 ± 0.20 ^b,b^	4.67 ± 0.35 ^c,b^	5.11 ± 0.16 ^b,a^
SLN20, G30, XG	0.41 ± 0.01 ^b,a^	0.02 ± 0.00 ^b,a^	39.50 ± 7.79 ^b,a^	0.34 ± 0.00 ^b,b^	0.01 ± 0.00 ^c,b^	44.96 ± 6.94 ^b,a^	0.33 ± 0.01 ^b,b^	0.01 ± 0.00 ^b,b^	51.36 ± 1.89 ^b,a^
SLN60, G30, CMC	1.95 ± 0.05 ^a,a^	1.52 ± 0.19 ^a,a^	14.29 ± 0.91 ^a,b^	1.28 ± 0.24 ^b,b^	0.65 ± 0.20 ^b,b^	20.87 ± 1.12 ^a,a^	1.50 ± 0.07 ^b,b^	1.01 ± 0.07 ^ab,b^	19.89 ± 0.88 ^b,a^
SLN20, G10, CMC	2.71 ± 0.19 ^ab,a^	0.43 ± 0.01 ^ab,a^	26.10 ± 1.37 ^a,a^	1.69 ± 0.09 ^a,b^	0.22 ± 0.01 ^a,b^	22.17 ± 3.05 ^b,a^	1.42 ± 0.38 ^ab,b^	0.21 ± 0.01 ^b,b^	25.44 ± 1.44 ^b,a^
	35 °C								
SLN60, G10, XG	5.41 ± 0.46 ^b,a^	10.56 ± 0.77 ^a,a^	3.47 ± 0.30 ^a,a^	5.73 ± 0.69 ^b,a^	11.39 ± 1.58 ^a,a^	4.64 ± 0.55 ^a,a^	5.13 ± 1.22 ^b,a^	7.26 ± 0.16 ^b,b^	4.17 ± 0.54 ^c,a^
SLN20, G30, XG	1.13 ± 0.02 ^a,a^	0.054 ± 0.01 ^a,a^	67.15 ± 8.39 ^a,b^	0.64 ± 0.05 ^c,b^	0.028 ± 0.00 ^b,b^	48.37 ± 2.69 ^b,c^	0.61 ± 0.06 ^b,b^	0.03 ± 0.00 ^a,b^	93.23 ± 5.00 ^a,a^
SLN60, G30, CMC	1.72 ± 0.53 ^a,a^	1.41 ± 0.22 ^a,a^	14.31 ± 2.79 ^a,b^	0.37 ± 0.02 ^c,b^	0.026 ± 0.00 ^c,b^	100.68 ± 15.07 ^b,a^	1.13 ± 0.16 ^b,ab^	1.26 ± 0.34 ^a,a^	13.62 ± 0.86 ^a,b^
SLN20, G10, CMC	2.76 ± 0.32 ^b,a^	0.53 ± 0.08 ^a,a^	26.27 ± 2.80 ^a,b^	1.77 ± 0.05 ^a,b^	0.12 ± 0.04 ^b,b^	30.93 ± 2.18 ^a,ab^	1.97 ± 0.29 ^a,b^	0.53 ± 0.01 ^a,a^	34.54 ± 3.38 ^a,a^

Each value represents the mean ± standard deviation of three replicates. Different letters indicate statistically significant differences (*p* ≤ 0.05); the first letter corresponds to the analysis based on the temperature and the second to the RH of the storage.

**Table 5:** Total color difference (ΔE) and whiteness index (WI) of edible films under different storage conditions.

	ΔE	WI	ΔE	WI	ΔE	WI
Formulation	60% RH		70% RH		85% RH	
	10 °C					
SLN60, G10, XG	4.43 ± 1.06 ^b,a^	96.38 ± 0.28 ^a,a^	4.67 ± 0.65 ^b,a^	96.33 ± 0.23 ^a,a^	4.89 ± 0.36 ^b,a^	96.10 ± 0.37 ^a,a^
SLN20, G30, XG	3.34 ± 0.20 ^b,b^	95.65 ± 0.37 ^ab,a^	3.46 ± 0.11 ^b,b^	95.70 ± 0.43 ^a,a^	4.53 ± 0.06 ^a,a^	96.26 ± 0.09 ^a,a^
SLN60, G30, CMC	3.94 ± 0.34 ^b,b^	95.03 ± 0.17 ^a,b^	3.63 ± 0.33 ^b,b^	95.00 ± 0.18 ^a,b^	5.16 ± 0.18 ^c,a^	95.99 ± 0.19 ^a,a^
SLN20, G10, CMC	3.60 ± 0.29 ^b,a^	96.45 ± 0.56 ^a,a^	4.08 ± 0.58 ^a,a^	95.87 ± 0.08 ^a,a^	4.00 ± 0.67 ^a,a^	95.93 ± 0.18 ^a,a^
	25°C					
SLN60, G10, XG	6.21 ± 1.01 ^ab,a^	95.18 ± 0.12 ^b,a^	5.52 ± 0.73 ^b,a^	94.17 ± 0.54 ^b,ab^	5.84 ± 0.96 ^b,a^	93.71 ± 0.71 ^b,b^
SLN20, G30, XG	3.48 ± 0.27 ^b,b^	96.19 ± 0.36 ^a,a^	4.08 ± 0.76 ^ab,ab^	95.65 ± 0.46 ^a,a^	4.96 ± 0.63 ^a,a^	95.32 ± 0.41 ^b,a^
SLN60, G30, CMC	5.63 ± 0.13 ^a,b^	95.45 ± 0.16 ^a,a^	6.61 ± 0.95 ^a,ab^	95.10 ± 0.68 ^a,a^	7.93 ± 0.40 ^a,a^	94.49 ± 0.19 ^b,a^
SLN20, G10, CMC	4.01 ± 0.15 ^ab,a^	96.27 ± 0.31 ^a,a^	5.08 ± 0.76 ^a,a^	95.26 ± 0.77 ^a,ab^	4.86 ± 0.10 ^a,a^	94.82 ± 0.45 ^b,b^
	35°C					
SLN60, G10, XG	7.33 ± 0.36 ^a,a^	94.16 ± 0.39 ^c,a^	6.59 ± 0.33 ^a,b^	91.28 ± 1.12 ^c,b^	6.96 ± 0.13 ^a,ab^	92.72 ± 0.75 ^b,ab^
SLN20, G30, XG	4.82 ± 0.79 ^a,a^	94.76 ± 0.77 ^b,a^	5.26 ± 0.58 ^a,a^	94.82 ± 0.11 ^a,a^	5.04 ± 0.47 ^a,a^	94.79 ± 0.37 ^b,a^
SLN60, G30, CMC	6.28 ± 0.84 ^a,a^	93.70 ± 0.53 ^b,a^	6.61 ± 0.31 ^a,a^	93.90 ± 0.76 ^a,a^	6.44 ± 0.53 ^b,a^	93.80 ± 0.44 ^b,a^
SLN20, G10, CMC	4.92 ± 0.71 ^a,a^	95.34 ± 0.56 ^a,a^	4.89 ± 0.37 ^a,a^	93.76 ± 0.62 ^b,b^	4.90 ± 0.54 ^a,a^	94.55 ± 0.03 ^b,ab^

Each value represents the mean ± standard deviation of three replicates. Different letters indicate statistically significant differences (*p* ≤ 0.05); the first letter corresponds to the analysis based on the temperature and the second to the RH of the storage.

## Data Availability

The data presented in this paper are available upon request from the corresponding author.

## Abbreviations

ANOVA	Analysis of Variance
ASTM	American Society for Testing and Materials
a_w_	Water activity
BSE+BSE (U)	backscattered electron (U shape)
CMC	carboxymethyl cellulose
DLS	dynamic light scattering
DSC	Differential Scanning Calorimeter
E	elongation at breaking
FDA	U.S. Food and Drug Administration
GRAS	generally recognized as safe
PDI	polydispersion index
PS	particle size
PVA	polyvinyl alcohol
RH	relative humidity
SEM	Scanning Electron Microscopy
SLN	solid lipid nanoparticles
Tmax	maximum temperature
TS	tensile strength
WI	whiteness index
WVP	water vapor permeability
WVT	water vapor transmission
XG	xanthan gum
YM	Young’s modulus
ΔE	total color difference
ΔH	enthalpy change
ζ	zeta potential
